# Mechanical ventilation with lower tidal volumes does not influence the prescription of opioids or sedatives

**DOI:** 10.1186/cc5969

**Published:** 2007-07-13

**Authors:** Esther K Wolthuis, Denise P Veelo, Goda Choi, Rogier M Determann, Johanna C Korevaar, Peter E Spronk, Michael A Kuiper, Marcus J Schultz

**Affiliations:** 1 Department of Intensive Care Medicine, Academic Medical Center, University of Amsterdam, Amsterdam, The Netherlands; 2 Department of Anesthesiology, Academic Medical Center, University of Amsterdam, Meibergdreef 9, 1105 AZ Amsterdam, The Netherlands; 3 Laboratory of Experimental Intensive Care and Anesthesiology (LEICA), Academic Medical Center, University of Amsterdam, Meibergdreef 9, 1105 AZ Amsterdam, The Netherlands; 4 Department of Clinical Epidemiology and Biostatistics, Academic Medical Center, University of Amsterdam, Meibergdreef 9, 1105 AZ Amsterdam, The Netherlands; 5 Department of Intensive Care Medicine, Gelre Hospitals, location Lukas, Albert Schweitzerlaan 31, 7334 DZ Apeldoorn, The Netherlands; 6 HERMES Critical Care Group, Amsterdam, The Netherlands; 7 Department of Intensive Care Medicine, Medical Center Leeuwarden, Henri Dunantweg 2, 8934 AD Leeuwarden, The Netherlands

## Abstract

**Introduction:**

We compared the effects of mechanical ventilation with a lower tidal volume (V_T_) strategy versus those of greater V_T _in patients with or without acute lung injury (ALI)/acute respiratory distress syndrome (ARDS) on the use of opioids and sedatives.

**Methods:**

This is a secondary analysis of a previously conducted before/after intervention study, which consisting of feedback and education on lung protective mechanical ventilation using lower V_T_. We evaluated the effects of this intervention on medication prescriptions from days 0 to 28 after admission to our multidisciplinary intensive care unit.

**Results:**

Medication prescriptions in 23 patients before and 38 patients after intervention were studied. Of these patients, 10 (44%) and 15 (40%) suffered from ALI/ARDS. The V_T _of ALI/ARDS patients declined from 9.7 ml/kg predicted body weight (PBW) before to 7.8 ml/kg PBW after the intervention (*P *= 0.007). For patients who did not have ALI/ARDS there was a trend toward a decline from 10.2 ml/kg PBW to 8.6 ml/kg PBW (*P *= 0.073). Arterial carbon dioxide tension was significantly greater after the intervention in ALI/ARDS patients. Neither the proportion of patients receiving opioids or sedatives, or prescriptions at individual time points differed between pre-intervention and post-intervention. Also, there were no statistically significant differences in doses of sedatives and opioids. Findings were no different between non-ALI/ARDS patients and ALI/ARDS patients.

**Conclusion:**

Concerns regarding sedation requirements with use of lower V_T _are unfounded and should not preclude its use in patients with ALI/ARDS.

## Introduction

One recent and substantive advance in the field of intensive care medicine has been the clear demonstration by the ARDS Network investigators [1] of the benefit conferred by lung protective (LP) mechanical ventilation (MV) among patients with acute lung injury (ALI)/acute respiratory distress syndrome (ARDS). Specifically, LP MV with lower tidal volume (V_T_; 6 ml/kg predicted body weight [PBW]), as opposed to conventional MV using larger V_T _(12 ml/kg PBW), was found to result in significant reductions in mortality and morbidity in these patients. A recent study conducted by the same investigators [2] confirmed that use of use of lower V_T _is associated with a low mortality rate. Despite the impressive results of the ARDS Network trial, many intensive care units (ICUs) have been slow to adopt LP MV [3]. Among the reasons for not adopting LP MV was the concern that its use would necessitate or increase prescription of sedatives and opioids because of patient intolerance of lower V_T _and increased respiratory rate (RR). Two secondary analysis of the ARDS Network trial, however, have clearly shown that lower V_T _ventilation does not increase sedation requirements in the first few days after initiation of MV in patients with ALI/ARDS [4,5].

We recently proposed that LP MV be employed in all intubated and mechanically ventilated patients, irrespective of the presence or absence of ALI/ARDS [6]. There are several reasons not to separate patients with from those without ALI/ARDS. First, diagnosing ALI/ARDS is at times challenging [3]. Indeed, although the consensus criteria appear relatively simple to apply [7], use of higher levels of positive end-expiratory pressure can improve both the arterial oxygen tension (PaO_2_)/fractional inspired oxygen (FiO_2_) ratio and abnormalities on chest radiographs to the extent that the patients no longer have ALI/ARDS (by definition) [8,9]. Second, patients may not yet fulfill consensus criteria at the initiation of MV but they may develop ALI/ARDS during the course of their disease. Third, critically ill patients are at constant risk for lung injury from other causes (for example, ventilator-associated pneumonia and transfusion-related ALI). A multiple hit theory can be suggested, in which repeated challenges lead to the clinical picture of ALI/ARDS. Unfortunately, however, no studies have yet investigated sedation requirements in patients who are not suffering from ALI/ARDS.

The present analysis was performed for the following reasons. First, we wondered whether the adoption of LP MV would affect sedation requirements in our ICU, where a strict sedation protocol is applied that is aimed at achieving the lowest possible level of sedation [10,11]. Second, because we favor the use of LP MV using lower V_T _in all patients, irrespective of the presence or absence of ALI/ARDS, we were interested in whether sedation requirements change with the use of lower V_T _in patients who are not suffering from ALI/ARDS. Finally, because the ARDS Network protocol prescribed the use of lower V_T _throughout ventilation (also with spontaneous MV at later time points), we wished to determine the impact of lower V_T _on sedation requirements in patients for a longer period (not only during the first few days of MV).

## Materials and methods

This is a secondary analysis of consecutive patients included in an interventional multicentre study [12]. In this study we determined the effect of feedback and education on use of lower V_T _in intubated and mechanically ventilated patients. The study included patients managed using a conventional V_T _strategy (before feedback and education; conducted in June 2003) and patients ventilated using a lower V_T _strategy (after this intervention; performed in January 2004) in our ICU. Only patients who were intubated and mechanically ventilated for longer than 24 hours were included. The study protocol was approved by the local ethics committee; informed consent was not deemed necessary because of the retrospective observational nature of this study and because the study did not require modification to diagnostic or therapeutic strategies.

### Intervention

The intervention has been described previously [12]. It consisted of four components. The first component was a concise presentation to all ICU physicians on results from several animal studies [13,14] and clinical studies of LP MV using lower V_T _in ALI/ARDS patients [1,15-20]. The second component a reminder about what was stated in our local MV guideline on size of V_T _(V_T _should be 6 to 8 ml/kg PBW) and a reminder that we all agreed to use lower V_T _when this guideline was introduced. The third component was a presentation of data on the actual size of V_T _before this intervention ('feedback'), for which two of the investigators (EKW and MJS) recorded all ventilator settings in all patients over a period of 2 weeks. The fourth and final component was a discussion of potential reasons for not using lower V_T _(including the importance of using PBW instead of actual bodyweight to set V_T_) and potential concerns that lower V_T_ will increase the need for sedation to maintain ventilator synchrony and comfort ('education'). The same strategy was employed for the ICU nurse team.

This intervention was repeated three times. Finally, the patient data management system (PDMS; Metavision, iMDsoft, Sassenheim, The Netherlands) was equipped with a special tool that automatically calculated the ideal V_T _from patient's height, after which the targets were automatically generated in the 'respiratory tab' (for all patients it was easy to check whether V_T _was between 6 and 8 ml/kg PBW).

### Mechanical ventilation guideline

Our local MV guideline has previously been described [12]. In short, before the intervention the guideline stated that pressure controlled or pressure support MV should be used in all patients. The guideline advised use of V_T _between 6 and 8 ml/kg PBW. After the intervention, the guideline explicitly advised that LP MV be used with lower V_T _(6 ml/kg PBW). Of note, although it was mentioned in the guideline that a RR above 20 breaths/min was considered uncomfortable for patients before the intervention, after the intervention no statements were given regarding RR.

In our ICU, the pressure level with pressure controlled or pressure support was adjusted to achieve the target V_T_. Because it was unit policy that both nurses and physicians were able to adjust ventilatory settings on a hourly basis, 24 hours per day, V_T _settings were subject to frequent adjustment and refinement.

### Sedation guideline

In our institution, standard intravenous sedation consists of the combined infusion of morphine and midazolam with 50 ml syringes pre-filled with 50 mg midazolam plus 50 mg morphine in sterile saline or glucose. Propofol can be used in addition, when high dosages of morphine and midazolam are needed, or solely, when frequent neurological evaluation is warranted. Morphine can also be used separately to control pain when there is no further need for sedation. The goals of sedation are to reduce agitation, stress and fear; to reduce oxygen consumption (heart rate, blood pressure and minute volume are measured continuously); and to reduce physical resistance to and fear of medical examination and daily care.

**Figure 1:**
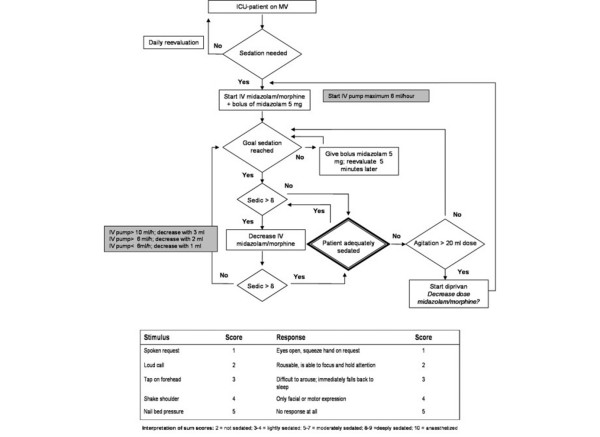
Diagram of the sedation protocol and SEDIC score. ICU, intensive care unit; IV, intravenous; MV, mechanical ventilation; SEDIC, Sedation Intensive Care.

According to the guideline (Figure 1), nurses and physicians determine the level of sedation required each day. Every 2 hours, the adequacy of sedation in each patient is carefully evaluated using a Sedation Intensive Care (SEDIC) score, and the infusion of sedatives is adjusted accordingly [10]. The SEDIC score consists of five levels of stimuli (from normal speech to nail bed pressure) and five levels of responsiveness (from normal contact to no contact). Sedation levels are defined by the sum of stimulus and response. When a SEDIC score above 8 is reached, infusion of sedation is reduced. In addition, patients weaned from midazolam receive low-dose oral benzodiazepines (lorazepam and temazepam). Haloperidol is given only to agitated or delirious patients.

### Clinical data collection

The following baseline data were extracted following initiation of MV: sex, body weight, height, admission diagnosis, and Acute Physiology and Chronic Health Evaluation (APACHE) II score. The diagnosis of ALI/ARDS was made by two investigators (EKW and MJS) using the consensus criteria for ALI/ARDS [7]. Ventilator settings were recorded at four different time points each day (08:00; 12:00, 18:00 and 24:00 hours) for a maximum period of 29 days. MV data (V_T_, maximum airway pressure [Pmax], positive en-expiratory pressure, RR, MV mode, FiO_2_, PaO_2 _and PaCO_2_) and sedation data (dose and timing of sedatives and opioids) were extracted from the PDMS, also for a maximum period of 29 days. Medication doses were recorded beginning on the day of MV initiation and ending at patients death, termination of MV, or day 29 of MV. Patients who were re-intubated within 24 hours remained in the analysis. Daily doses of morphine, midazolam and propofol were calculated. Doses included both intravenous boluses administered on an as-needed basis and continuous intravenous infusions. Sedation given for intubation or tracheotomy was not included in the calculations.

### Statistical analysis

Data are presented for the whole study period. Data are presented as mean ± standard deviation for normally distributed data, or as median (interquartile range) for data that are not normally distributed. Differences between two groups were assessed using a Mann-Whitney U-test or Student's *t*-test for continuous variables; differences between four groups were assessed using Kruskal-Wallis test or one-way analysis of variance. χ^2 ^test was used for categorical variables. Linear mixed model analysis was used to study changes over time in patients. This type of analysis takes into account the association between values for individual patients measured at each time point. This implies a maximum of 29 time points per patient. The fixed effects were day of MV (0 to 28), group (before or after feedback and education, and having ALI/ARDS or not) and the interaction between group and day of MV. Data obtained with linear mixed model analysis are presented as mean (95% confidence interval). Statistical calculations were done using SPSS 12.0.1 (SPSS Inc., Chicago, IL, USA). Differences with a *P *value < 0.05 were considered statistically significant.

## Results

### Patients

Baseline characteristics of 23 and 38 patients were collected before and after the intervention, respectively (Table 1). ICU mortality was 18% after the intervention as compared to 39% before the intervention (*P *= 0.08). The number of ventilation days was significantly greater in ALI/ARDS patients both before and after the intervention, as compared with non-ALI/ARDS patients. Also the PaO_2_/FiO_2 _ratio on admission was significantly lower in ALI/ARDS patients in both groups.

**Table 1:** Demographic variables

	Before intervention (*n *= 23)		After intervention (*n *= 38)		*P *value
	Non-ALI/ARDS (*n *= 13)	ALI/ARDS (*n *= 10)	Non-ALI/ARDS (*n *= 23)	ALI/ARDS (*n *= 15)	

Male (*n *[%])	9 (69)	4 (40)	14 (61)	9 (60)	0.55
ABW (kg; median [IQR])	70.0 (70.0 to 75.0)	75.0 (61.5 to 97.0)	76.0 (70.0 to 95.0)	70.0 (60.0 to 80.0)	0.25
PBW (kg; median [IQR])	64.2 (52.4 to 70.6)	57.5 (52.6 to 70.1)	69.7 (59.7 to 75.1)	66.9 (57.9 to 75.1)	0.19
APACHE II (mean ± SD)	20.2 ± 7.5	22.8 ± 11.3	21.8 ± 7.1	19.9 ± 6.4	0.76
MV^a ^(days; median [IQR])	4.0 (3.0 to 11.5)	13.5 (6.5 to 26.0)	5.0 (2.0 to 8.0)	13.0 (5.0 to 22.0)	0.004
ICU death (*n *[%])	2 (15)	7 (70)	3 (13)	4 (27)	0.005
PaO_2_/FiO_2 _ratio^b ^(median [IQR])	225 (194 to 264)	138 (120 to 157)	216 (173 to 268)	146 (100 to 187)	< 0.001
Admission diagnosis (*n *(%])					
Medical	2 (15.4)	1 (10)	2 (8.7)	7 (46.7)	
Surgical	2 (15.4)	2 (20)	5 (21.7)	4 (26.7)	
Neurology/neurosurgery	5 (38.5)	2 (20)	6 (26.1)	2 (13.3)	
Cardiopulmonary surgery	2 (15.4)	3 (30)	3 (13.0)	2 (13.3)	
Cardiology	2 (15.4)	2 (20)	6 (26.1)	0	

*P *values were determined using χ^2 ^test or one-way analysis of variance, comparing all four groups. ^a^Total number of mechanical ventilation (MV) days during the study period. ^b^On admission. ABW, actual body weight; ALI, acute lung injury; APACHE, Acute Physiology and Chronic Health Evaluation; ARDS, acute respiratory distress syndrome; PBW, predicted body weight.

### Mechanical ventilation

V_T _was significantly lower after the intervention (Table 2). V_T _in ALI/ARDS patients declined from 9.7 ml/kg PBW before to 7.8 ml/kg PBW after the intervention (*P *= 0.007); for non-ALI/ARDS patients there was a trend toward a decline, from 10.2 ml/kg PBW to 8.6 ml/kg PBW (*P *= 0.073). Accordingly, RR increased significantly in ALI/ARDS patients after the intervention (*P *< 0.001). In addition, RR in non-ALI/ARDS patients after the intervention was higher than that in ALI/ARDS patients before the intervention (*P *= 0.049). For non-ALI/ARDS patients the RR did not increase after the intervention. PaCO_2 _was significantly greater in ALI/ARDS patients after the intervention than in non-ALI/ARDS patients (*P *= 0.017) and ALI/ARDS patients (*P *= 0.034) before the intervention. Pmax did not differ between the two study periods. However, Pmax was significantly greater in ALI/ARDS patients than in non-ALI/ARDS patients after the intervention (*P *= 0.031).

**Table 2:** Respiratory variables for the whole period of 29 days

	Before intervention (*n *= 23)		After intervention (*n *= 38)		*P *value
	Non-ALI/ARDS (*n *= 13)	ALI/ARDS (*n *= 10)	Non-ALI/ARDS (*n *= 23)	ALI/ARDS (*n *= 15)	

V_T _(ml/kg PBW)	10.2 (9.3 to 11.1)	9.7 (8.9 to 10.5)	8.6 (7.8 to 9.5)	7.8 (7.2 to 8.5)	<0.001
Pmax (cmH_2_O)	18.2 (13.0 to 23.4)	21.8 (18.1 to 25.5)	16.8 (11.4 to 22.2)	25.6 (22.8 to 28.5)	0.01
RR (breaths/min)	21.3 (18.4 to 24.3)	18.3 (16.1 to 20.6)	23.4 (20.5 to 26.4)	25.6 (23.7 to 27.6)	<0.001
PaCO_2 _(kPa)	5.0 (4.3 to 5.7)	5.3 (4.8 to 5.8)	5.5 (4.9 to 6.2)	6.2 (5.8 to 6.6)	0.007

*P *values were determined using linear mixed model analysis, comparing all four groups. Values are expressed as mean (95% confidence interval). ALI, acute lung injury; ARDS, acute respiratory distress syndrome; PaCO_2_, partial pressure of arterial carbon dioxide; Pmax, maximum airway pressure; RR, respiratory rate; V_T_, tidal volume.

### Prescription of sedatives and opioids

**Figure 2:**
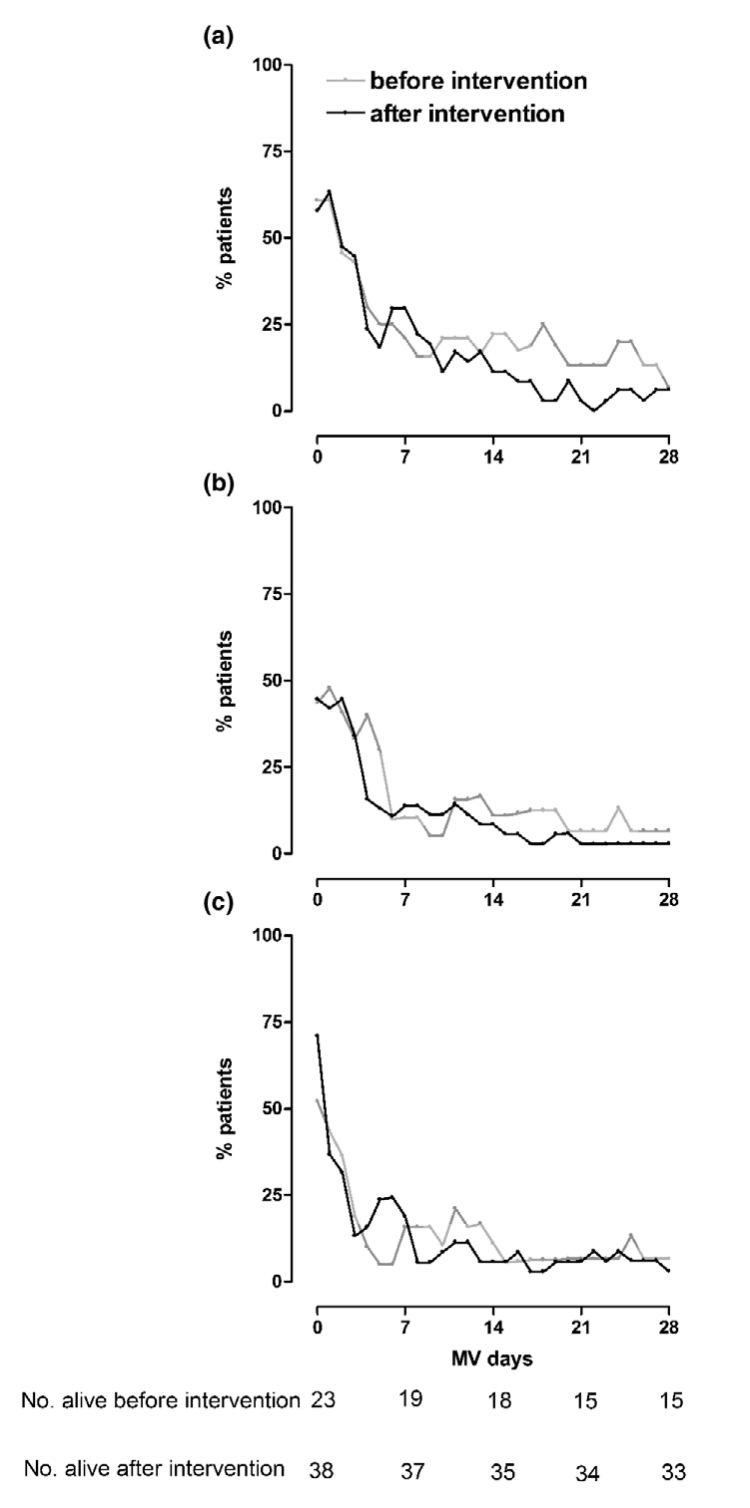
Percentage of patients requiring sedative drugs. Shown are the percentages of patients needing **(a) **morphine, **(b) **midazolam, or **(c) **propofol from days 0 to 28 for patients mechanically ventilated before and after the intervention.

The mean doses of morphine, midazolam, or propofol for mechanically ventilated patients on days 1, 7, 14, 21 and 28 are presented in Table 3. The percentages of mechanically ventilated patients requiring morphine, midazolam, or propofol for these time points are presented in Table 4. There were no significant differences in terms of doses of morphine, midazolam, or propofol before and after the intervention. Also, there were no significant differences in the percentage of mechanically ventilated patients needing morphine, midazolam, or propofol. Figure 2 shows the percentage of patients (either mechanically ventilated or liberated from the ventilator) needing morphine, midazolam, or propofol during the study period. There were no differences for sedatives or opioids at any time point.

**Table 3:** Dosing of opioids and sedative drugs at different time points

	Before intervention	*n*	After intervention	*n*	*P *value
	
Morphine					
Day 1	0.54 ± 0.68	23	0.70 ± 0.85	38	0.55
Day 7	0.64 ± 0.99	11	0.49 ± 0.66	20	0.74
Day 14	0.53 ± 0.67	7	0.65 ± 0.72	8	0.90
Day 21	0.88 ± 0.78	3	0.06 ± 0.13	5	0.21
Day 28	0.25 ± 0.36	2	0.50 ± 0.81	3	0.67
Midazolam					
Day 1	0.65 ± 1.0	23	0.84 ± 1.3	38	0.94
Day 7	0.82 ± 1.9	11	0.69 ± 1.8	20	0.80
Day 14	0.32 ± 0.58	7	1.7 ± 3.5	8	0.45
Day 21	0.40 ± 0.70	3	3.8 ± 8.6	5	0.42
Day 28	0.37 ± 0.52	2	6.0 ± 10.4	3	0.45
Propofol					
Day 1	19.4 ± 35.3	23	13.5 ± 22.6	38	0.64
Day 7	3.0 ± 8.2	11	13.9 ± 29.8	20	0.49
Day 14	10.6 ± 27.8	7	21.8 ± 49.2	8	1.0
Day 21	24.6 ± 42.5	3	52.3 ± 97.6	5	0.60
Day 28	7.9 ± 11.2	2	32.0 ± 55.4	3	0.63

Data are presented in mg/kg per day as mean ± standard deviation. The *P *value was derived using the Student's *t*-test.

**Table 4:** Number of patients needing opioids and sedative drugs

	Before intervention	*n*	After intervention	*n*	*P *value
Morphine					
Day 1	14 (61)	23	24 (63)	38	0.86
Day 7	4 (36)	11	11 (55)	20	032
Day 14	4 (57)	7	4 (50)	8	0.78
Day 21	2 (67)	3	1 (20)	5	0.19
Day 28	1 (50)	2	2 (67)	3	0.71
Midazolam					
Day 1	11 (48)	23	16 (42)	38	0.61
Day 7	2 (18)	11	5 (25)	20	0.66
Day 14	2 (29)	7	3 (38)	8	0.71
Day 21	1 (33)	3	1 (20)	5	0.67
Day 28	1 (50)	2	2 (67)	3	0.71
Propofol					
Day 1	10 (43)	23	14 (38)	38	0.66
Day 7	3 (27)	11	7 (35)	20	0.66
Day 14	2 (29)	7	3 (38)	8	0.88
Day 21	1 (33)	3	1 (20)	5	0.67
Day 28	1 (50)	2	1 (33)	3	0.71

Values are expressed as number (%). *P *values were determined using χ^2 ^test.

### ALI/ARDS patients versus patients without acute lung injury

We used linear mixed model analysis to determine whether there were changes over time before and after the intervention, and whether the presence versus absence of ALI/ARDS affected the use of morphine, midazolam, or propofol. When patients were subdivided into ALI/ARDS patients and non-ALI/ARDS patients, there was no difference in the mean dose of sedative drugs for the different groups (Table 5). The percentage patients with ALI/ARDS needing morphine over the entire study period was 70% before and 93% after intervention (*P *= 0.12). The respective percentages were 80% and 87% for midazolam (*P *= 0.66), and 90% and 93% for propofol (*P *= 0.76).

**Table 5:** Sedative drugs for the whole period of 29 days

	Before intervention (*n *= 23)		After intervention (*n *= 38)		*P *value
	Non-ALI/ARDS (*n *= 13)	ALI/ARDS (*n *= 10)	Non-ALI/ARDS (*n *= 23)	ALI/ARDS (*n *= 15)	

Morphine					
Mean (mg/kg per day; 95% CI)	0.18 (-0.33 to +0.69)	0.61 (0.25 to 0.97)	0.11 (-0.41 to +0.64)	0.61 (0.33 to 0.90)	0.18^a^
Patients needing morphine (*n *[%])	10 (77)	7 (70)	18 (78)	14 (93)	0.49^b^
Midazolam					
Mean (mg/kg per day; 95% CI)	0.08 (-1.8 to +1.64)	0.24 (-1.15 to +1.62)	0.04 (-1.60 to +1.69)	2.04 (0.93 to 3.15)	0.08^a^
Patients needing midazolam (*n *[%])	8 (62)	8 (80)	10 (43)	13 (87)	0.04^b^
Propofol					
Mean (mg/kg per day; 95% CI)	0.88 (-20.4 to +22.2)	14.5 (-0.99 to +23.0)	5.96 (-16.4 to +28.3)	19.3 (7.12 to 31.5)	0.44^a^
Patients needing propofol (*n *[%])	9 (69)	9 (90)	20 (87)	14 (93)	0.30^b^

CI, confidence interval. ^a^*P *value for comparison of means by linear mixed model analysis, comparing all four groups. ^b^*P *value for comparison of number of patients by χ^2 ^test. ALI, acute lung injury; ARDS, acute respiratory distress syndrome.

## Discussion

Because preservation of neurological function is critical to the accurate identification of clinical improvement or deterioration in critically ill patients, sedation requires careful consideration [21]. Prolonged sedation increases utilization of unnecessary diagnostic studies [22], and it may lead to delayed extubation, lengthening of the stay in the ICU and worsen clinical outcomes [22,23]. High-dose, continuous sedation can also decrease long-term quality of life [24]. Concerns that the need for sedation will be increased by the use of LP MV with lower V_T _are not supported by the findings of the present study; after implementing this strategy in our ICU, patients did not require higher levels of sedation or opioids as compared with patients managed before implementation of the strategy.

There are several limitations to our study. First, it was an observational cohort study. Over recent years there has been growing awareness of the benefits of restrictive use of sedatives. One may suggest that increased sedation requirements may be masked by this improved awareness. We believe that this time-dependent effect plays a minor role, because the two periods of data collection are close together and our sedation guideline did not change during the conduct of this study. However, because we have a strict sedation guideline at our ICU, it may be possible that we did not observe influences on sedation requirements between the two groups, in particular because both ICU physicians and nurses are very stringent in applying the sedation guideline. Because of this, and perhaps also because of other factors that are not easy to recognize and that are unique to a single ICU, our results may not be applicable to other ICUs. Third, the total number of patients included in this study is quite small, especially when we subdivide patients into those who have ALI/ARDS and those who do not. It is therefore possible that type II errors (false-negative results) occurred and that subgroup analysis is unreliable.

However, our findings are quite similar to those of a study conducted by Cheng and coworkers [4]. In this secondary analysis of the ARDS Network trial, no differences were found between patients in the lower V_T _strategy and patients in the conventional V_T _strategy in terms of the need for sedation or neuromuscular blockade within 48 hours after admission. Kahn and colleagues [5] reported a similar analysis and found that there were no significant differences in the percentage of study days during which patients received sedatives, opioids, or neuromuscular relaxants (over a maximum period of 28 days). Our data extend the findings of these two studies by showing that the same applies to patients who are not suffering from ALI/ARDS.

One important finding is that after intervention the mean V_T _was 8.6 ml/kg PBW for non-ALI/ARDS patients and 7.8 ml/kg PBW for ALI/ARDS patients (and not 6 ml/kg PBW, as was the case in the ARDS Network trial [1]). Thus, the levels of V_T _in our study, after the intervention, are better considered to be 'intermediate' volumes rather than 'low' volumes. In fact, it must be recognized that the V_T_s are still high, and possibly too high in both patient groups. However, V_T _settings in patients suffering from ALI/ARDS declined during the conduct of this study. Nevertheless, the difference between V_T _before the intervention and that after the intervention is small, which be why we did not observe a difference between the two ventilation groups in terms of need for sedation.

## Conclusion

A decline in V_T _in ALI/ARDS patients at our center did not increase sedation requirements. For non-ALI/ARDS patients there was a trend toward a decline in V_T _from 10.2 ml/kg PBW to 8.6 ml/kg PBW (*P *= 0.073). In these patients there was also no increase in sedation needs. Concerns regarding the potential adverse effects of LP MV should not preclude its use.

## Key messages

• Lower V_T _did not increase sedation needs in ALI/ARDS patients and non-ALI/ARDS patients in our ICU.

## Abbreviations

ALI = acute lung injury; ARDS = acute respiratory distress syndrome; FiO_2 _= fractional inspired oxygen; ICU = intensive care unit; LP = lung protective; MV = mechanical ventilation; PaO_2_ = arterial oxygen tension; PBW = predicted body weight; Pmax = maximum airway pressure; RR = respiratory rate; SEDIC = Sedation Intensive Care; V_T _= tidal volume.

## Competing interests

The authors declare that they have no competing interests.

## Authors' contributions

EKW collected and analyzed the data. DPV, GC and RMD collected the data. JC helped with the statistical analysis. PES and MK reviewed the study. MJS reviewed and coordinated the study.
